# *Me?* The invisible call of responsibility and its promise for care ethics: a phenomenological view

**DOI:** 10.1007/s11019-018-9873-7

**Published:** 2018-10-16

**Authors:** Inge van Nistelrooij, Merel Visse

**Affiliations:** University of Humanistic Studies, Kromme Nieuwegracht 29, 3512 HD Utrecht, The Netherlands

**Keywords:** Care ethics, Responsibility, Phenomenology, Marion

## Abstract

Care ethics emphasizes responsibility as a key element for caring practices. Responsibilities to care are taken by certain groups of people, making caring practices into moral and political practices in which responsibilities are assigned, assumed, or implicitly expected, as well as deflected. Despite this attention for social practices of distribution and its unequal result, making certain groups of people the recipient of more caring responsibilities than others, the passive aspect of a caring responsibility has been underexposed by care ethics. By drawing upon the work of the French phenomenologist Jean-Luc Marion, a care ethical conceptualization of responsibility can by enriched, by scrutinizing how responsibility is literally a *response* to something else. This paper starts with a vignette of an everyday situation of professional care. After that the current body of care ethical literature on responsibility is presented, followed by Marion’s phenomenology of givenness, using his analysis of Caravaggio’s painting *The Calling of St. Matthew* and resulting in his redefinition of responsibility. In the next section we present a table in which we juxtapose four distinct paradigms of responsibility, which we will describe briefly. The final section consists of an exploration of the paradigms by an analysis of the vignette and results in a conclusion concerning what Marion’s view has to offer to care ethics with regard to responsibility.

## Prelude

‘On our surgery ward of a general hospital an elderly male patient is being transferred after surgery from the hospital to a nursing home for further revalidation. It was an emotionally charged event for him, since he was not sure if he would ever be able to return to his own home, after revalidation. So this transfer could be one not to a temporary home, but to his last home, the home in which he would live the last phase of his life.

His bag was packed, he was dressed and sitting in his wheelchair when his wife and daughter came to pick him up. When they arrived and the moment of his leaving the ward, his shoes seemed to have disappeared. I have been looking everywhere for his shoes, together with his wife and daughter, when suddenly it occurs to me that another patient was also transferred that same morning. Perhaps he might not only have taken his slippers but also this man’s shoes. Immediately I went to make a phone call and I turned out to be right. The family of this other patient discovered the accidentally taken pair of shoes and promised to bring them to the nursing home on the following morning, since their own affairs did not allow them to take immediate action in this case. I was quite satisfied and relieved with this solution.

When I returned to the patient who still waited for his shoes, telling him the good news, to my surprise he started to cry. It appeared that for the very first time in his 76 years he would have to go outside wearing only socks and this was especially hard since this was the special occasion of moving to his new home, what could be his final home. He would have to make his entrance there in this way, not fully dressed, lacking decorum, and he regretted it more than he could say. So I thought that I had solved a problem, but to him it was no solution to what he faced.’

The researcher asks: ‘What did you do next?’
Nurse: ‘Well, nothing. I could not do magic and let the shoes appear, could I?’
Researcher: ‘So what did you do next?’
Nurse: ‘I sat with the patient for a minute, took his hand, and said that I regretted it so much that he was sad because he had no shoes. He quieted down a little. We put a blanket over his legs that covered his feet as well.’

(The story of an elderly care nurse in: Van Nistelrooij 2008)

## Introduction

Care ethics emphasizes responsibility as a key element for caring practices (Gilligan 1982; Noddings 1984; Tronto 1993, 2013; Held 2006; Walker 2007; Kohlen 2009; Van Heijst 2011; Heier 2016; Van Nistelrooij 2015; Visse et al. 2015; Visse and Abma 2018). If responsibility is not taken or accepted, caring does not occur. Responsibilities to care are however taken by certain groups of people, for else nobody and nothing would be taken care of. Despite the lack of political and ethical attention for this everyday practice, our society would not exist without care. Margaret Walker and Joan Tronto especially clarify how social and political practices are practices in which these responsibilities are unequally distributed. Walker (2007) argues that people collectively express what they consider valuable in the way that social practices of responsibility are constructed; simultaneously these means of expression construct practices of responsibility by reproducing or shifting the terms of recognition (p. 10). People learn about their own identity, their relationships and the socially recognized values “through practices of responsibility in which they assign, accept or deflect responsibilities” (p. 10). What people do, collectively, in a moral sense, is constrained and made intelligible by the collective ideas about individual and social responsibilities. For instance, and drawing upon Van Heijst (2005, 2011), even though there may not be an official obligation for women to quit their job or cut back hours in order to take care of their children, cultural expectations for them to do so are persisting. Tronto (1993, 2013) problematizes the distribution of responsibilities, as this division tends to “privilege those who are excused by not needing to provide care; thus the privileged avoid responding directly to the actual processes of care and the meeting of basic needs” (1993, p. 121; cf. 2013, Chap. 2). In these views, responsibility is about having the task to respond to a need; this task can be explicitly assigned, e.g. to professionals, or implicitly expected from certain groups of people due to cultural patterns and hierarchical structures of power and status in society. And many people accept them, otherwise no society could exist and no people survive.

However, looking at responsibility as something that one ‘has’, as something that can be determined and delineated, and distributed among people, may also be problematic in a certain way. Even though the care ethical analyses have stressed that responsibilities cannot be looked upon apart from the social practice and political context in which they are distributed among people, we believe that this context may also cover the ambivalence of the immediate *experience* of responsibility. If responsibilities are primarily viewed as matters of social distribution in which power is also involved, other aspects of responsibility are left aside. Tronto (1993, 2013) has already shown that such a distributive view of responsibilities tends to make them personal, which is a way to marginalize and contain caring practices. Her analysis uncovers the harmful effects of such an individualizing and apolitical view and underpins her plea for a democratic account of care, making care a collective responsibility. We concur with her critique on this personalized view of responsibility as it creates inequalities and puts too much pressure upon certain individuals to meet their responsibilities and execute their caring task. In this paper, we also concur with the care ethical political-ethical analysis of power in which many care ethicists (Tronto 1993, 2013; Kittay 1999; Hankivsky 2004; Walker 2007; Held 2015; Visse et al. 2015) argue for care to be given its proper role in morality and politics. However, these pleas mainly focus upon the agents that respond to the need of care. If we truly aim to understand why care requires responsibility, we need to analyze not only how care is organized and performed in society, but we need to think through how a caring responsibility is *literally a response* to something else. We need to analyze how the caregiver as an agent is also a passive recipient of a need, a call for help, that can be overwhelming. This part of caring practices requires a phenomenological view of responsibility. Therefore, we turn to French phenomenologist Jean-Luc Marion’s view on responsibility.

Our purpose is to decrease the pressure upon individual caregivers’ responsibility by presenting an analysis of the *phenomenon of responsibility*. In order to do so, in Part A of this paper we briefly present the current body of literature on responsibility in the interdisciplinary field of care ethics. In Part B we present Jean Luc Marion’s phenomenological view of givenness using his analysis of Caravaggio’s painting *The Calling of St. Matthew*, followed by his analysis and redefinition of responsibility. In Part C, we will connect Marion’s view on responsibility with the current paradigms in care ethics on responsibility. In Part D, we will further explore the paradigms by an analysis of the vignette above and explore what his view has to offer for care ethical dimensions of the caring relationship.

### Part A: Care ethical accounts of responsibility

In care ethical theory several insights have proven to be critical touchstones for ethics, such as relationality, affectivity, contextuality and practices (Leget et al. 2017). With regard to a central concept like responsibility (Tronto 1993; Walker 2007), care ethics has emphasized the concreteness of responsibilities (Tronto 1993), the responsibility that emerges through dependency in prior relationships or situatedness (Kittay 1999), and as distributed in social practices (Walker 2007). The emphasis has been put on the responsibilities that are accepted or deflected by the caregiver, as a response to another person’s need or concern (Tronto 1993). A further scrutiny of dependency as the source for moral obligation, not only by the directly involved caregivers, but also by multiple others and society as a whole in support of caregivers, has been the lifework of Kittay (e.g. 1999, 2011). She has pointed out that a person giving care to extremely dependent persons (she uses the term dependency worker) needs to be transparant when giving care, so that the needs and interests of the dependent can be leading (Kittay 1999, p. 52). She argues that the caregiver in this situation needs to suspend her own plans and projects and be led by altruism (p. 52). However, the focal point of her analysis is still the caregiver’s and society’s moral agency, on what they are obligated to do.

Responsibility within care ethics not only co-determines the performance of good caregivers, as a characteristic of their involvement with another person’s need or concern and of their caring response, it is problematic too. For it may lead to a boundless commitment to others that is harmful to the caregiver’s self. This double-sided view is recognizable in how many professional caregivers discuss their responsibility as both defining and delineating their obligations in their role as caregivers. In this role a continuous search for taking *and* limiting responsibility takes place and professionals tend to warn each other especially for exceeding their limits (e.g. in familiar expressions like: “This is not your responsibility”, or “You should not exceed your responsibilities”, or “You should not take your responsibilities home with you”).

Part of the problems with responsibility has to do with care ethics’ roots in feminism. Feminist analyses point out that responsibility is never entirely a moral individual’s free choice. This leads care ethicists to acknowledge how caring tasks and responsibilities are unequally distributed and assigned (e.g. Walker 2007; Tronto 1993, 2013), how they arise in existing relationships (Kittay 1999), how social practices establish positions of ‘privileged irresponsibility’ and ‘caring passes’ on the one hand and marginalized positions of those involved in caring responsibilities on the other (Tronto 1993, 2013), how this distribution of responsibilities is connected to social and cultural ideas and ideals of freedom, success, privilege and autonomy as opposed to bondage, failure, subservience and dependency (Tronto 1993, 2013; Van Heijst 2011), and how they are always in the literal sense a response to a need or concern of somebody one meets, in whatever setting. So care ethics does not overestimate the caregiver’s agency but always considers it as bound up with social practices, cultural patterns, and *ethos*. A caregiver is never considered to be an entirely self-deliberating, self-determining, contract-signing, alternatives-weighing, and freely choosing person in care ethics. Rather caregivers appear as people finding themselves in a position in which others, they themselves, and also the socio-political context *expect them* to have and take responsibility, as a result of socio-political, personal, affective, contextual, and ethical factors. Their position is determined not only by their own contingency (the time, place, and web of relations in which they were born and raised), but also by their participation in collective practices within the family, school, work place, and by socio-political determinants like their age, gender, ethnicity, class, which co-determine their social status and power(less) position.

Nevertheless, there are nuances in how passivity is incorporated in care ethical views of the moral agent. In general, more politically oriented care theorists stress the powerless position of caregivers and their passivity in the sort or amount of caring responsibilities assigned to them (Walker 2007; Tronto 1993, 2013). They stress the need for empowerment of those involved in care, thereby emphasizing caregivers’ agency in political and moral respect (Tronto 1993, 2013; Engster and Hamington 2015; Held 2015). Walker (2007) has argued that whenever we shift our attention from the idea of ‘vulnerability-in-principle’ as a shared human condition in which actual dependency is an abstract possibility, in order to turn to real-life ‘dependency-in-fact’ (p. 90), we come to acknowledge that some people are concretely and acutely dependent upon others, and others are not. This distinction founds Walker’s proposal that a *geography of responsibilities* should be at the heart of ethics, since ‘tracking the lines of responsibility’ uncovers the social distribution of actual responsibilities and ‘who gets to do what to whom and who is supposed to do what for whom’ (p. 16).

In the same vein, Tronto (1993) considers the concrete caring practice of responding to needs as connected to its socio-political context. Caring is an ongoing practice that starts with the identification of a need, which entails that one ‘cares about’ something. This identification requires attentiveness, but if one leaves it at that, care does not come about. The caring practice needs to evolve into ‘taking care of’, that is the assumption that something needs to be done that requires the self to become involved. Therefore responsibility needs to be assumed with regard to a need:

Taking care of […] involves assuming some responsibility for the identified need and determining how to respond to it. Rather than simply focusing on the need of the other person, taking care of involves the recognition that one can act to address these unmet needs. […] Taking care of involves notions of agency and responsibility in the caring process (Tronto 1993, p. 106).

The emphasis here is on the caregiver as moral agent, actively involving herself in the process of addressing a need, as the heart of responsibility. It would be naive, however, to think of the moral agent as lacking the political context of power. For those who are involved in caring, are often those lacking privileges and power. Hence the key problem of a moral theory of care often is *not* the problem of moral motivation, but rather to reflect upon the ongoing practices of care in which they are enmeshed and look honestly at how sacrifices are unequally distributed and how caregivers’ situation and position are often not improved by the caring practices in which they are enmeshed (p. 141). Inequality is a real societal problem that is revealed when we look at the political context of care. Accepting caring responsibilities, then, may lead to further marginalization and loss of power.

Other care ethicists pay more attention to the passivity and responsiveness that is required of the caregivers by caring practices themselves (Noddings 1984; Kittay 1999; Van Heijst 2011). They strongly reject a view of care as a commodity or a practice that can be controlled. Instead they advocate a view of caring as an unpredictable relational practice of attuning to another unique human being in which the caregiver needs to let go of a pre-determined responsibility (Van Heijst 2011). Noddings (1984) describes caring as acting ‘from the position of one-caring’ (8), being led ‘not by fixed rule but by affection and regard […] varied rather than rule-bound […] with special regard for the particular person in a concrete situation’ (24). Others also acknowledge the unpredictable nature of caring practices and promote a view that centres moral learning as a practice of reflection on how responsibilities are distributed (Visse and Abma 2018). Even though no care ethicist argues for a complete lack of self-determination by the caregiver, many argue that care involves a form of temporary self-abandonment and being-led by the care receiver, whether it is called transparancy and altruism (Kittay 1999), engrossment and motivational displacement (Noddings 1984, 2002, 2015), or self-sacrifice (Van Nistelrooij 2015).

Van Heijst (2011) developed yet another stance on responsibility, and her view is called upon here for two reasons. First, she explicitly specifies her view to the *professional* caregiver’s responsibility and takes institutional settings into account. And second, her view of responsibility describes the *responding character* of responsibility that we explore in this contribution. She positions the specific professional caregiver’s responsibility at the crossroad of the professional’s skill of knowing how to help and of being able to transform a situation of need, on the one hand, and their proximity to patients and clients who need them, on the other (p. 177). She develops a multi-layered idea of professional responsibility in which ability and moral obligation go hand in hand, since knowing how to help entails that you should. What is more, she includes the ways in which responsibility is ascribed to persons, making them feel responsible, which is also connected to professionals having consciously taken the position as a healthcare professional in the nearness of patients and clients. Thereby they are simultaneously held responsible by others and feeling responsible themselves (p. 177). Her analysis leads her to conclude that on a fundamental level of human neediness, all human beings are dependent upon each other for their mere survival, but also for being able to lead a meaningful life (p. 145). This is a fundamental human equal neediness that we share as human beings and should be recognized as communality. Yet on the level of the concrete situatedness people are unequally dependent because there is an evident inequality of need (p. 144). “Evident asymmetry characterizes care situations and that should not be hidden.” (pp. 144–145) Hence Van Heijst concludes to a double structure of neediness: one on the surface, of evident unequal needs, and one on a deeper level, of equal neediness as humans. This double structure of neediness leads Van Heijst to conclude that a mixture of feelings, cognition and mediating institutional and professional regulations is required for professional responsibility:

[P]rofessionals need feelings, reasoning and an institutional context of regulations that modifies and facilitates their taking moral responsibility. The head is a complementary guide to the heart, and ideas of institutional obligation help to do what professionals believe is best (Van Heijst 2011, pp. 196–197).

In each of these positions a mediated form of responsibility takes centre stage. Each care practitioner is formed by the position of one-caring (Noddings), practices (Walker), the socio-political context (Tronto), relations of dependency (Kittay), and the institutional context of health care (Van Heijst), in which the caregiver has formed and internalized ideas, ideals, expectations, norms, and regulations. Yet this practitioner also has the freedom to make moral decisions, to answer to or ignore moral appeals. Responsibility still appears as moral task, as something one *has or has not*, and can decide about. This thought fits perfectly with the idea that freedom is required for morality, for without freedom (e.g. when coerced) one cannot be held responsible. But it also acknowledges givenness: that the caregiver is called upon, that a need is there that needs to be responded to. This aspect can be enriched by Marion’s phenomenology, as we will argue in the next section. From a phenomenological stance, however, the subject does not decide, but receives much more than one could ever imagine (2002, p. 309).

### Part B: Marion’s phenomenology of responsibility as givenness

When Marion looks at responsibility in *Being Given* (2002), this is what he sees (pp. 282–296).^1^ First there is a call. Like in Caravaggio’s painting *The Calling of St Matthew* that is on the book’s cover, one cannot see a caller or the call itself but only Matthew, making the silent gesture of the left hand pointing to his chest, indicating the question ‘Me?’. With this gesture the only thing that is visible is the reception of a call by Matthew. This picture says a lot about responsibility according to Marion (and as will be clear to those readers familiar with Emmanuel Levinas’ work, Marion’s view draws upon Levinas’ work in important ways). It shows ‘in silence a call that is invisible’ (283), as the call comes from the outside of the subject, but also from outside the subject’s horizon. In the picture there is only a light shining upon Matthew from outside the picture’s frame. This ‘coming from the outside’ to Marion means that the call is not preceded by expectation or by a ‘hearing’ capability, since that would be a metaphysically set horizon of a subject able to receive. Such a presupposed horizon of ability limits the possibilities for phenomena to appear: only what is visible to the eye, what is audible to the ear, etc. can count as a phenomenon. By opposing such a limitation, Marion positions himself as a phenomenologist who aims to radicalize Husserlian phenomenology. For Husserl the appearing of phenomena still happens to a consciousness, i.e. intentionality. Marion concurs with other phenomenologists who have criticized Husserl for this centrality of (human) intentionality and who stress the fact that not everything can be grasped by consciousness. In fact, as long as we can determine the conditions for what appears, phenomena cannot appear as they are. Therefore Marion argues:

Appearing must thus remove itself from (if not always contradict) the imperial rule of the a priori conditions of knowledge by requiring that what appears force its entry onto the scene of the world, advancing in person without a stuntman, double, or any other representative standing in for it (Marion 2002, p. 69).

With this rich imagery Marion aims to show how already grasped reality, through experiences, mastered knowledge and gathered images, may reduce the possibility of phenomena to appear and to be received. Radicalizing the phenomenological openness requires the breaking through of our horizon and the entrance of a call from the outside (c.f. the light coming from outside the painting’s frame).

What is more, this light in the painting may in itself be open to many interpretations, but for Matthew its meaning seems immediately obvious (Marion 2002, p. 285): it calls him and catches him in surprise by the look of his face. Some of the persons next to him seem to recognize that something is happening here, pointing at him too, others look up in wonder and the light shines on their faces too, still others do not notice what is going on. In a quite literal sense Matthew is depicted here as a screen on which the light shines, making it visible. Matthew looks up and nonverbally asks: ‘Me?’ This is his response and this response makes him visible as the one *to whom has been given*, the one who Marion renames as *gifted* (p. 287).

The appearance of the phenomenon of the call, underpinned by Caravaggio’s painting, is deconstructed by Marion as distinguishable aspects that precede responsibility. First there is the invisible call (p. 283). This call can be regarded as a manifestation in itself and by itself that lends itself to its reception (p. 287). But the call itself is not a visible signal; rather it remains indistinct and outside the picture’s frame, outside any subjective horizon of hearing and seeing. We can only see the call in the second ‘stage’, i.e. its manifestation in which it becomes visible in Matthew’s figure that makes the call visible, which Marion calls the *responsal* (pp. 287–288, emphasis in text).

By admitting itself to be the target of the call, therefore by responding with the simple interrogative “Me?” the gifted opens a field for manifestation by lending itself to its reception and the retention of its impact (p. 287).

The *responsal* is the first response of the gifted, but ‘nothing like an optional act, an arbitrary choice, or a chance’ (p. 288), for the call is not pulled into a subject’s horizon but rather the gifted (the one to whom has been given) lets the call speak, lets the call phenomenalize (p. 288). Here the gifted is like a prism, a screen, on which the phenomenon becomes visible, and like the prism or the screen, the gifted has nothing to say about what becomes visible (p. 288). And third, after that, there may (or may not be) a belayed response. This third ‘stage’ leading to responsibility is the belated response that is the transforming effect upon the gifted. The reconceptualizations of the subject and of responsibility are intertwined, as the following quotation shows:

All the determinations by which the phenomenon gives *itself* and shows *itself* starting from *itself* to the point of exerting a call [...] are concentrated and transcribed for the gifted in the responsibility that he suffers from them. The pertinent question is not deciding if the gifted is first responsible toward the Other (Levinas) or rather in debt to itself (Heidegger), but understanding that these two modes of responsibility flow from its originary function of having to respond in the face of the phenomenon as such, that is to say, such as it gives itself (Marion 2002, pp. 293–294).

Here we find his *redefinition of responsibility*. It is a ‘having to respond’ that should not be understood as a will that decides about seeing or hearing. Such an understanding would be a metaphysical idea of the will that is opposed by Marion (p. 305). The subject in his view does not decide, but the given (phenomenon) humbles the gifted (p. 305), as the gifted receives much more than one could ever imagine (p. 309). And since responsibility is a matter of (involuntarily) receiving the given (i.e. the phenomenon), this moment is not preceded by any vision or reason. Rather, the gifted ‘suffers from having to respond in the face of the phenomenon’, Marion states (p. 294), and this suffering consists in the opening of a space that has been unforeseen and unimagined and is therefore filled with fright (p. 306):

Here opens a space of indecision that cannot be imagined without fright: the decision in favor of staging the given as a phenomenon, therefore also that in favor of the reason of things, can be made only without vision or reason since it makes them possible. The responsal decides with nothing other than itself alone (p. 306).

In Marion’s rich linguistic terms, relating to grammar: the subject is not in the nominative, nor in the accusative (like with Levinas). Rather, the subject is in the dative: he or she is ‘the gifted’, i.e. the “unto whom/which” (p. 249), or the one to whom has been given. Here we see the passivity of responsibility in its extreme version: the given gives itself and the gifted receives the self as gifted at the same time (p. 308). Hence the self has the chance to see the self in an overwhelming new space that has come to existence by the shattering of one’s previous horizons. Through givenness the self has been given the chance to cross one of the greatest divides that he can cross: his indifference (p. 308).

Concluding this first presentation of Marion’s redefinition of responsibility as radical givenness, we argue that this radical phenomenology offers a profound view of responsibility’s passive aspect that is not lacking in care ethics, but has been underexposed. In what follows we aim to discern between the various views of responsibility and see what Marion’s thoughts may add.

### Part C: Four paradigms for thinking responsibility

When we explore the various conceptualizations and meanings that have been given to responsibility, we may disclose beforehand the four paradigms that will emerge from this exploration (see Table. 1). The first two paradigms draw upon an analysis and argumentation by care ethicists Eva Feder Kittay (1999) and Margaret Urban Walker (2007), the third paradigm draws upon Walker as well, but places it in a socio-political context that involves moral learning (Landeweer 2018; Visse et al. 2012). The fourth paradigm entails Marion’s view of givenness (Table 1).

**Table 1:** Four paradigms for thinking responsibility

Voluntaristic moral obligations	Vulnerability-responsive moral obligations	Expressive-collaborative moral obligations	Radical givenness
Paradigm: the promise	Paradigm: the other’s vulnerability to my actions	Paradigm: social routines and expectations (effected through identities and relations)	Paradigm: involuntarily having to respond to a call entering my ear
Moral basis = the obligation that I assume when I give my word	Moral basis = the needs of another	Moral basis = individually and socially practiced normative expectations	Moral basis: the appearance of a phenomenon
Obligation = – To whomsoever I made a promise – Self-assumed, free choice – I must fulfill my promise	Obligation = – Arises out of the relationship between one in need and one who is situated to meet the need – Is situational: when I am in the special position vis-à-vis the other to meet the needs	Obligation = – Arises out of the social negotiation process between people in practices who undergo those same practices	No pre-existing or arising obligation Responsibility is a response to a call; the call is nothing more and nothing less than the given opportunity to abandon of indifference
Problems 1. Ignores non-voluntary obligations in existing relations 2. Presupposes a society of equally situated and empowered individuals	Problems 1. Presupposes a ‘pragmatic ought’ that ignores coercion 2. Ignores how someone comes to be in a position where another is vulnerable to one’s actions	Problems 1. Not everyone has the means to participate in negotiations (be ‘at the table’) 2. Not everyone has negotiation ‘skills’ and to relate to/resist the powerful normative social practices 3. Assignment of responsibilities is based on implicit assumptions	Problems Ignores existing power structures, cultural and structural mediation, and bonds of the flesh
View of the subject – Detached, autonomous agent	View of the subject – Permeable, dependent, relational subject	View of the subject – Participant in socio-political and relational practices – Participant in asymmetric relationships (power)	View of the subject – Amoral or premoral recipient – The gifted – Decentered subject

The first paradigm considers responsibility as something that has been accepted freely, like in a promise (Kittay 1999), that can be determined from a moral standpoint outside practices (Tronto 1993; Van Nistelrooij et al. 2014), and fits within a theoretical-juridical view of morality (Walker 2007). The underlying anthropological assumption is that all moral agents are equal as individuals with equal rights (‘individual-based equality’, Kittay 1999) and that moral questions therefore centre around which rights I have as an individual and what rights others may assert. The emphasis is on the moral agent as a free, unconnected, detached individual, who is both capable of and in the position to voluntarily and autonomously assuming an obligation (or not). Kittay draws upon Robert Goodin’s work *Protecting the Vulnerable* (1985) when she calls this the voluntaristic model and contrasts it to the vulnerability model (Kittay 1999, pp. 54–55). In this voluntaristic model a moral obligation is not only voluntarily assumed, the obligation is also limited in two ways, viz. to the one to whom the promise is made and to the promise’s content, hence to do that (and only that) which I promised (p. 55). This model becomes even more clear when one looks at what is left out by these restrictions: the agent acting according to this model does not owe anything to others (a person, a group of persons, nor society as a whole) that he has not committed himself to. No pre-existing relationship or community like a family or a community leads to a moral obligation that can be forced upon him or her without allowing the freedom of the agent to decide about it by making a well-considered deliberation on interests, values, norms and aims. The only foundation that is shared with others is the expectation that anyone who incurs an obligation freely by making a promise, honors such a promise (p. 55).

The second paradigm is rooted in a different understanding of what it means to be human, of morality, and of moral epistemology. This paradigm takes into account social practices in which power is always a determining factor. This paradigm first of all acknowledges connections: everybody is equal, because everybody has come to be somebody because somebody else has taken care of (‘mothered’) him or her, as expressed by Kittay in her famous quote: ‘We are all—*equally*—some mother’s child’ (Kittay 1999, p. 25, emphasis in text). Hence we are equal as human beings because of our connectedness (‘connection-based equality’, Kittay 1999, p. 66). Moral questions are intertwined with this connectedness, since we find ourselves in a network of relations in which concrete responsibilities are distributed, accepted and deflected through social practices (Walker 2007).

These responsibilities are central issues in the moral questions that are asked in this paradigm: what are my responsibilities and normative expectations towards others and what are those of others towards me? Kittay profoundly analyses these questions when she considers the moral nature of dependency relations (Kittay 1999, pp. 49–73). Since in this paradigm vulnerability and dependency are acknowledged as characteristics of the human condition, moral obligations arise in existing relationships and in situations in which the moral agent is in the position to help (Kittay 1999, p. 55). On the one hand power can be discovered in these practices, as some can demand of others to fulfil their responsible tasks. On the other hand, power also serves as a ‘lens’ through which we can look at these practices, asking ourselves how some come to be in the position in which they have to care for others, even if this is at the cost of their own self-interest or wellbeing (Tronto 1993; Kittay 1999; Walker 2007). These questions ask for a socio-political view of care, that Kittay develops by assigning responsibilities and moral obligations not only to the ones giving care, but also to those receiving care and to society as a whole. The latter two have to take into account that caregivers are vulnerable too. This makes care recipients—to the best of their abilities—obliged to avoid damage for those taking care. And since the caregivers’ vulnerability results from their suspended interests, the affective bond, and their concern for the charge’s wellbeing, social support is needed too, so that care does not become a liability to one’s own wellbeing (Kittay 1999, pp. 65–66).

To conclude with regard to the second paradigm, responsibilities are rooted in the human condition of vulnerability. And since we all share this vulnerability, society as a whole depends upon some to take care of all of us. Therefore care is not limited to interpersonal and temporary caring relationships, but these should be nested within broader caring relations that embrace the needs of each, and as such care as a practice is the foundation of our society. Or to put it in Kittay’s own words:

For the dependency worker to meet her responsibilities to another, it must be the responsibility of the larger social order to provide a structure whereby she, too, may be treated as a mother’s child (1999, p. 70).

The third paradigm draws upon Walker’s collaborative-expressive view on responsibility practices. Basic is the idea that morality is not disconnected from social practices: in an interplay of social practices and one’s participation, one gains ‘moral understandings’ (Walker 2007) on one’s identity, relations and the values that are shared socially. In social practices some identities (characterized by gender, class or ethnicity) have necessarily and ideologically been linked to servitude or care (Walker 2007, pp. 153–176), and even if the ideological foundation of these necessary identities is no longer valid nowadays, the connected cultural expectations have not disappeared (Van Heijst 2005, p. 305). As a result, moral agents are consistently confronted with social environments that expect them to either take or refrain from certain responsibilities, and deviations from these patterns of responsibilities are characterized by a tension between social context and individual decisions.

Walker’s view of morality as collectively expressed has been placed in the context of a socio-political learning process (Landeweer 2018; Visse et al. 2012). Responsibilities are ‘negotiated’ through a mutual learning process of people where they aim to understand what matters to them, why it matters and what their underlying commitments are (Visse and Abma 2018). People find themselves in practices of ‘mutually allotting, assuming or deflecting responsibilities’ (ibid, p. 67). They continuously attune, interpret and anticipate responsibilities, either consciously or implicitly, and are subject to power asymmetries while ‘negotiating’ on their perspectives. In this view on responsibility, moral expectations are not fixed and context free, but fluid, intertwined with place, time, the stage of their practice, identities and the nature and quality of relationships. Despite the meticulous attention for facilitating conditions for learning and negotiation (Visse et al. 2015; Landeweer et al. 2010), the problem with this view is that the subject is still assumed as an agent, able to articulate or ‘capture’ what matters in particular practices through thorough research.

Furthermore, in all these three paradigms, moral agents appear as subjects who can reject their assigned responsibilities—even if they are subjected to a social, cultural and political context which leaves them little room for critical reflection upon their social and moral position. In these models, however, little attention is paid to the pre-reflexive realms of experience. These three paradigms can be labeled as ‘ontic’ ways of looking at responsibilities. Epistemologically, we can ‘know’ responsibility practices by analysis of what occurs in the reality ‘out there’, like geographers mapping responsibilities. A different, phenomenological view on perception and thinking about responsibilities would be attentive to our pre-reflexive, implicit dimensions of experience. This is what the fourth paradigm is grounded upon. It draws upon Marion’s phenomenological view that has been illustrated at the start of our analysis. In comparison to the previous paradigms, his view sheds light on a different, ontological dimension and introduces the ‘givenness’ of responsibility. Both in the voluntaristic and in the vulnerability-responsive paradigm, the focus of attention is on the moral agent. Whether one assumes or rejects responsibilities; whether one has been assigned or coerced into the responsible position for answering to the needs of others; or whether one’s responsibilities result from the interplay of powerful normative social practices and negotiation, responsibilities are primarily taken as something *one owns and has to do*. Differing from these paradigms, the focus of Marion’s analysis draws our attention to the passive side. Here the moral agent first of all is not a moral agent, but an amoral or premoral recipient of a sign, a signal, an insight, a call, or whatever forceful sight or sound it is that forces itself upon the self. This self is the gifted, who is not an agent yet. In other words: the relationality of the self is stressed to the point where one is no longer ‘one’, but always secondary to phenomena, to what arises, and to an invisible call. For first there is a phenomenon that appears rather than a subject that thinks, sees, and hears. As we saw, Marion is especially interested in opening the horizon that in much philosophy is set by this *ego* of the Cartesian *cogito*. This *ego* of consciousness, awareness and (Husserlian) intentionality still has a containing effect on phenomena, preventing the appearance of phenomena that cannot be grasped, take us by surprise, are beyond our imagination and exceed intelligence.

However, phenomenology and such language (horizon, appearance of phenomena, surprise, exceeding intelligence) may be strange to ethicists. We are aware that we need to construct an argumentative connection between ethics and this radical phenomenology. We believe that our view of responsibility gains by including this passively receiving aspect as it puts the subject into perspective in two ways. First, the subject is no longer the centre of responsibility but understood through *what has been given*, and this understanding decreases the burdensome centrality of a caregiver’s position. The decrease consists in the self’s shift that is illustrated by Marion in linguistic terms: a shift from the first person’s perspective (nominative) to the third person’s perspective (dative), meaning that responsibility starts elsewhere, not foreseen, unexpected, and is received as affect. As such it invites, it gives the possibility to respond to a call, to abandon indifference, to enter a commitment to a need. Second, Marion’s view offers the possibility of an increased understanding of the caregiver’s situation, since it suspends the normative and thereby allows for a non-judgmental look. Through the de-centring of the subject and allowing for phenomena to appear from a non-normative stance, a more complex reality of caring practices can be understood. Especially with regard to a concept like responsibility, that is so often referred to in an individualizing normative sense (‘you should have taken the responsibility to…’) this seems appropriate. It helps to refrain from simplification and blame.

Hence Marion’s reconceptualization of responsibility must not be understood in a moral sense. A subject is more than a moral person and responsibility is also something that Marion aims to disconnect from morality first and analyse in a phenomenological way. So Marion’s work is that of a radical phenomenologist who deliberately disconnects phenomena and the self from ethics. Therefore, his phenomenology is focused upon phenomena appearing as themselves, which he strongly emphasizes, rather than on ‘how they are experienced’ or ‘how they are performed’. To give an example: in an article on love and charity (Marion 1994) he expressly stipulates that these phenomena should not be ‘devaluated’—as he calls it—by making them objects of ‘making or doing’: ‘making love’ and ‘doing charity’ are expressions that ‘prostitute’ and ‘betray’ the phenomena ‘love’ and ‘charity’ as phenomena (ibid, p. 168). In other words: it must be possible to look at ‘love’ and at ‘charity’ as appearing phenomena in themselves, to discover what occurs, other than a tool, act or aim of an agent. In the same vein we claim that responsibility *as phenomenon* can be understood as the ongoing appearance of a call for responsibility; and refrain from any objectification (prostitution, betrayal) of responsibility by connecting it to verbs like ‘accepting, assuming, deflecting’, which in turn presuppose a subject, and a subject’s horizon.

### Part D: Connecting the paradigms with the vignette

From the point of view offered by the voluntaristic model (paradigm 1), the nurse’s responsibility for the shoes is non-existing, and her actions are best characterized as something supererogatory. She never explicitly promised or implicitly committed herself in any way to take care of the shoes. Perhaps the nurse’s hospital (like probably many if not all hospitals in and outside the Netherlands) has explicitly rejected any responsibility or liability for patients’ belongings. So her search for the shoes, the arrangements she makes, let alone the time she gives to the patient in his distress, are at most morally admirable, but in no way her responsibility.

From the point of view of Kittay’s vulnerability-responsive model (paradigm 2), the nurse was rightly affected by the patient’s vulnerability for travelling without shoes. She was not only present when the disappearance of the shoes was discovered, she was also in the best position to do something about it. She had knowledge of the previously transferred patient and she had access to his personal information. Hence her position allowed and required her to take adequate action. However, Kittay’s model extends the moral obligation. It is not only about the action owed by the best situated actor, but it requires a caregiver (or dependency worker in her vocabulary) to become transparant and responsive to the charge’s concern, since this—and primarily and exclusively this—should be leading in care. So the nurse may be acting responsibly, her response is not responsive since it at first lacks openness for the ‘real’ problem. As a parallel to Kittay’s story of caregiving to her daughter (‘Not my way, Sesha. Your way. Slowly.’ Kittay 1999, pp. 157–161), giving care is not about doing things the way that the caregiver wants, feels urged, is obliged, is pressed or prescribed to do. Rather, giving care is about *giving oneself over* to the other. In this story, Sesha’s caregiver Peggy tries very hard to do walking exercises with Sesha, Kittay’s daughter with severe mental retardation and cerebral palsy. But these efforts are in vain, since Sesha appears to be completely occupied with something else. Sesha’s eyes are fixed on the falling leaves in Central Park (p. 157). This is what ‘vulnerability-responsiveness’ is all about: one needs to know what the charge is (most) concerned with. And care needs to be attuned to that, therefore the caregiver *as a caregiver* is required to be altruistic, i.e. be transparant to the charge’s needs, and not have them blocked or refracted by one’s own needs (p. 52).

To fully understand the assignment of responsibilities in the case from Walker’s perspective (paradigm 3), we would search for more information on past experiences and routines of the nurse and the patient and his wife and daughter, as Walker assumes that personal identities and their histories are constitutive for the assignment and acceptance of responsibilities. Previously we argued that the nurse could have acted responsibly, but she was not responsive to the real problem (the man having to leave without shoes). However, what expectation was beyond the man’s crying? Before the nurse can accept responsibility, we should—in line with the ‘vulnerability-responsive’ model—be open to what the other’s (moral) needs are. Her response, to cover his legs with a blanket, focused on a social solution, because the man experienced a lack of decorum and didn’t want to be seen on his socks by other people. But we may wonder if this was merely a social issue. Maybe he didn’t want to walk on socks for himself, to protect his personal dignity. Next, maybe his need for a ‘proper’ entrance into his new home could have been responded toward in other ways as well. At this point, we don’t know. In order to fully understand the case example from Walker’s perspective, we would need to contextualize the case example (‘thicken’ it with narrative) in order to fully understand the practice of responsibility that unfolded in this particular care. We would, for example, also inform about how the nurse and the patient got to know each other, how their relationship looked like and what routines developed between them (as compared to other caring relationships in this setting) during the time the patient was hospitalized. We would reconstruct the case by including the dialogue between the nurse and man. Next, we would be open to learn about the socio-political context of the residential home: this could relate to the way the nurse responded to the patients’ need and whether and how she usually accepts responsibility for the patient’s needs. Thus, this paradigm would be interested in the context and historicity of their relationship, as well as any possible opposing factors outside the context of their relationship (e.g. institutional policies).

Now if we turn to Marion’s radical phenomenology (paradigm 4) we can see that when the nurse becomes aware of the patient’s actual need, she becomes a different kind of agent. She expresses this very well herself. She says that her arrangements have not comforted the patient, but have rather deepened his sorrow and sadness. When asked ‘what did you do next’, she literally says: ‘Nothing, I couldn’t do magic and let the shoes appear, could I?’ And when the researcher asks again: ‘So what did you do next?’ she speaks of her reception (first) and consequent response to the patient’s concern. She sits down with him, makes physical contact (taking his hand), expresses how she was affected by the sadness that she received from him (‘[I] said that I regretted it so much that he was sad’) and this response has the effect that he quiets down. And then there is also a practical solution: the covering of the feet with a blanket. What we see here, is a nurse first trying to take care of a problem. As soon as the shoes appear to be missing, she starts acting. When she thinks that she has arranged an adequate solution, and therefore has answered to her responsibility, she appears to be wrong. When asked what she did when meeting the patient’s disappointment, she says: ‘nothing’. But what she is actually left with after this moment, is the reception of the man’s call. Now his need ‘enters’ her, causing her to open up, to abandon her active mode, and become responsive. She turns into a ‘passive’ receiver and thus makes herself a ‘vessel’ of the responsibility that can live through her and her relationship with the man and others, even though activities are involved (sitting down, taking his hand, expressing regret). This passivity consists in her reception of his emotions, of his bodily presence, of everything that is contained in that particular situation. Even though at the start of the vignette, she had received his practical need for his shoes which prompted her into action, here she receives in a different mode. Her receptivity is no longer on the practical level of ‘correcting mistakes’ and trying to accomplish good care for this patient, but rather on the level of what it *means* for *this* patient. *What it means to him* is expressed and seeks a receiver, lends itself to be listened to, to affect another, inviting an affective rather than an active response. This call is not so much a call expressed by the man verbally. Instead, it is a call that is pre-reflexive, beyond our knowing, grounded in the being of that situation itself.

## Conclusion

Marion’s view has something to offer to care ethics that can be summed up as follows: first, he offers a view of the passive, receptive dimension of human existence and also of responsibility; second, his view widens the horizon of responsibility in caring practices and shows that within these practices much more is given and received that transcends anyone’s responsibility. Both of these points help to decrease the emphasis on moral obligation of the moral agent by putting agency into perspective. Agency, we propose, stands in a dialectic tension with passivity, and this tension illuminates how not everything depends upon the caregiver as a moral agent. We believe that this dialectic view is helpful to caregivers as they can emerge as both agents *and* recipients of responsibility, in an ongoing movement.

We have illustrated that a phenomenological analysis of responsibility, based on the work of Jean-Luc Marion, points towards a passive side of care: a responsible person or society *responds* to something or someone, and hence there is something coming first, uncontrolled by the one who ‘merely’ receives it. There is a ‘re-active’ dimension to responsibility, that seems to make sense in caring practices: one becomes responsible for something or someone by receiving a ‘call’. This call appears within a relational practice with someone *who is in some kind of need for care*. This—what we call—passive dimension of responsibility, in which the caregiver first receives something (a hint, a signal, a sound, a view, a smell, a silence), disentangled from her own morality, moral ideal, or ethical aim, has not yet received much attention in care ethics. We hope this paper inspires care ethicists to further explore the meaning of this.

## Footnotes

1 For this analysis we draw upon Van Nistelrooij (2015).
